# Emphysematous Pyelonephritis Involving Native Kidneys and a Transplanted Kidney

**DOI:** 10.7759/cureus.29024

**Published:** 2022-09-11

**Authors:** Venu Chippa, Swetha Chenna, Rahul Gujarathi

**Affiliations:** 1 Internal Medicine, St. Vincent Medical Center, Evansville, USA; 2 Internal Medicine, Indiana University Bloomington, Bloomington, USA; 3 Hospital Medicine, University of Florida Health, Jacksonville, USA

**Keywords:** emphysematous cystitis, septic shock, transplant kidney, immunocompromised, emphysematous pyelonephritis

## Abstract

Emphysematous pyelonephritis (EPN) is an acute severe necrotizing form of UTI, with air collection in the renal parenchyma, collecting system, and surrounding tissues. Most common causative organisms are *Escherichia coli and Klebsiella*. Here we present a 71-year-old Caucasian male with multiple medical problems who was transferred to our ER from a long-term acute care hospital (LTACH) for hematuria, UTI, and septic shock. He is a recipient of a deceased donor renal transplant and was receiving IV amphotericin B for recent cryptococcal meningitis. CT abdomen showed bilateral EPN, including the transplanted kidney. He received aggressive fluid resuscitation, vasopressors, and IV antibiotics and got admitted to the ICU. Unfortunately, his clinical course worsened and required ventilatory support, and his family opted for comfort care because of the high mortality rate. The emphasis of this case report is to investigate immunocompromised patients presenting with UTIs for EPN.

## Introduction

Emphysematous pyelonephritis (EPN) is a rare and severe form of acute necrotizing UTI, with air collection in the renal parenchyma, collecting system, and surrounding tissues. The first case of gas-forming necrotizing renal infection was reported in 1898 by Kelly and MacCallum. The direct and indirect cost of acute pyelonephritis is estimated at around 2.5 billion US dollars (the year 2000 values) in the US [1]. The pathogenesis of EPN is multifactorial. Compromised renal perfusion and urinary retention are major precipitating factors in the development of EPN. *Escherichia coli* is the most common causative organism, followed by *Klebsiella* sp., *Proteus* sp., *Enterobacter* sp., and rarely *Streptococcus* sp. and *Candida* sp. Diabetes mellitus, kidney stones, and immunocompromised state are significant risk factors. 

With the current trends of increasing incidence and prevalence of diabetes mellitus and frequent use of abdominal imaging for abdominal discomfort associated with UTI, more and more cases of emphysematous cystitis (EC) and EPN are being diagnosed [2]. Women are predominantly affected with recurrent UTIs, and so as EPN, the female-to-male ratio is 5: 1. Left side is most commonly affected [3]. Bilateral EPN is reported in about 10% of patients [4]. The mortality rates in EPN are high, with about 50% with medical management alone and 15% even with surgical intervention/percutaneous drainage [5]. Septic renal transplant patients have threefold higher mortality than uncomplicated UTIs [6]. 

Even though EPN is commonly reported in radiology and urology journals, currently, internists provide most of the care for hospitalized patients, so we suggest EPN be in differential diagnoses while caring for diabetes and immunocompromised patients with complicated UTIs. Here we report a case of EPN involving both native and transplanted kidneys. To our knowledge, this is the first-ever reported case of EPN in the medical literature where all three kidneys are involved.

## Case presentation

A 71-year-old Caucasian male was transferred from a long-term acute care hospital (LTACH) to our hospital with significant hematuria, UTI, and low blood pressure with suspected septic shock. About a month ago, he was diagnosed with COVID-19 pneumonia and disseminated cryptococcal infection, and is currently on amphotericin B. Past medical history is significant for a cerebrovascular accident with aphasia requiring tracheostomy and percutaneous endoscopic gastrostomy (PEG) tube placement, insulin-dependent diabetes mellitus with hemoglobin A1c of 8.6%, a deceased donor renal transplant five years ago on the right side, and recurrent extended-spectrum beta-lactamase (ESBL) *E. coli* UTI. His current medications are prednisone, azathioprine, tacrolimus, aspirin, atorvastatin, furosemide, pantoprazole, folic acid, insulin, and amphotericin B. 

The patient is nonverbal at baseline from his old stroke. In the ER, his blood pressure was 75/41 mmHg, temperature 36.6 °C, pulse rate 105 beats per minute (bpm), respiratory rate 26 per minute, and oxygen saturation of 96% on room air. On examination, he appeared in no distress, patent tracheostomy tube with bilateral basal crackles on both lung fields, normal S1-S2 with no murmur, soft and non-tender abdomen with faint bowel sounds, gastrostomy tube with the normal surrounding skin, was able to follow simple commands, and capillary refill time of 3-4 seconds with no extremity edema was noticed. Foley catheter showed cloudy and blood-tinged urine. 

**Figure 1:**
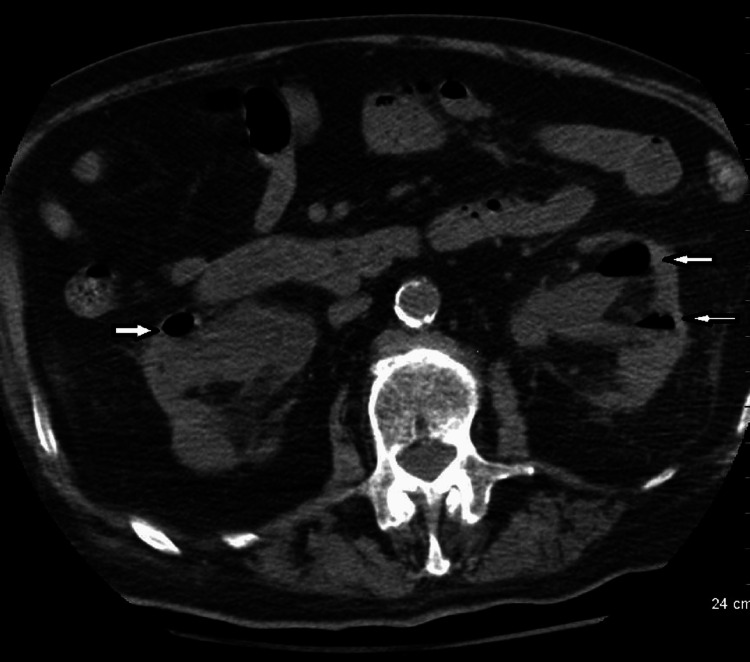
Non-enhanced CT image showing gas in both the right and left kidneys (white arrows).

**Figure 2:**
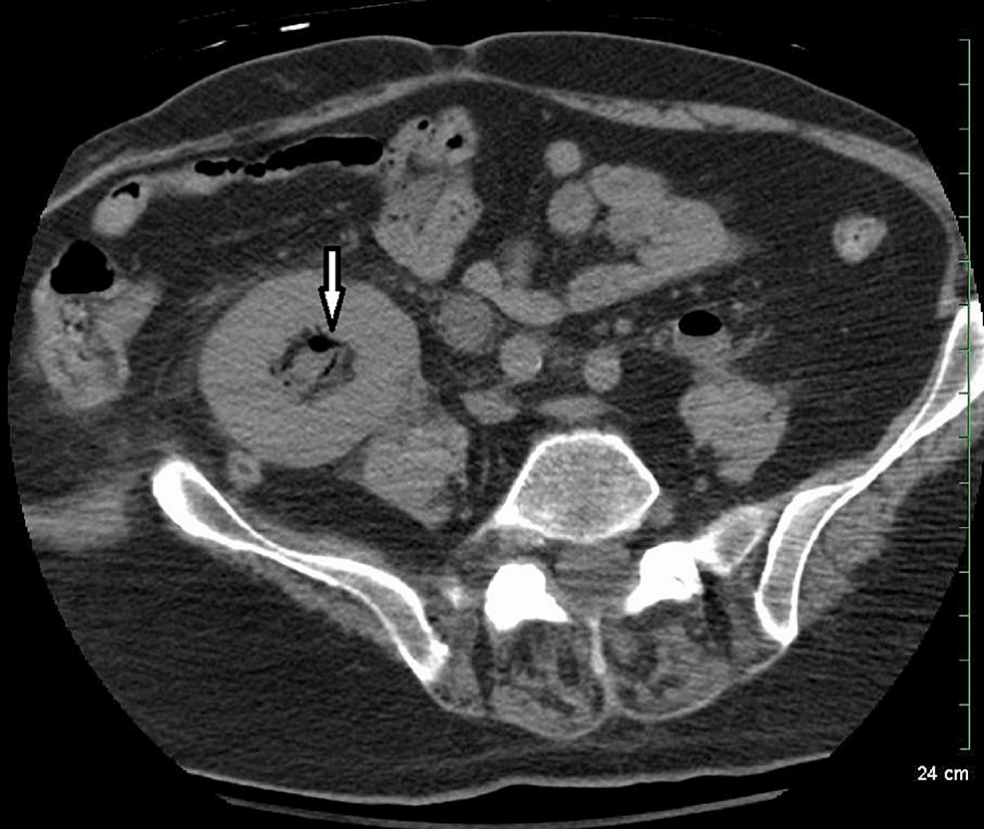
Non-enhanced CT image showing gas in the transplanted kidney pelvis (white arrow).

**Figure 3:**
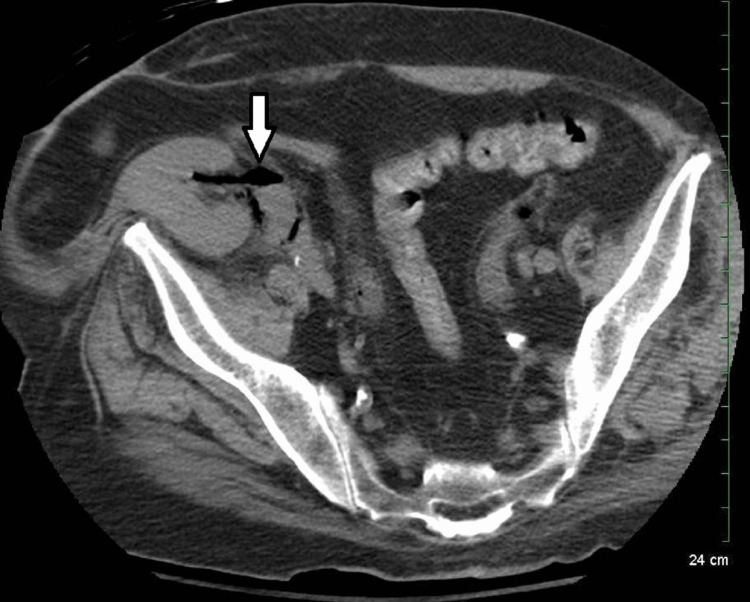
Non-enhanced CT image showing gas in the transplanted kidney proximal ureter (white arrow).

**Figure 4:**
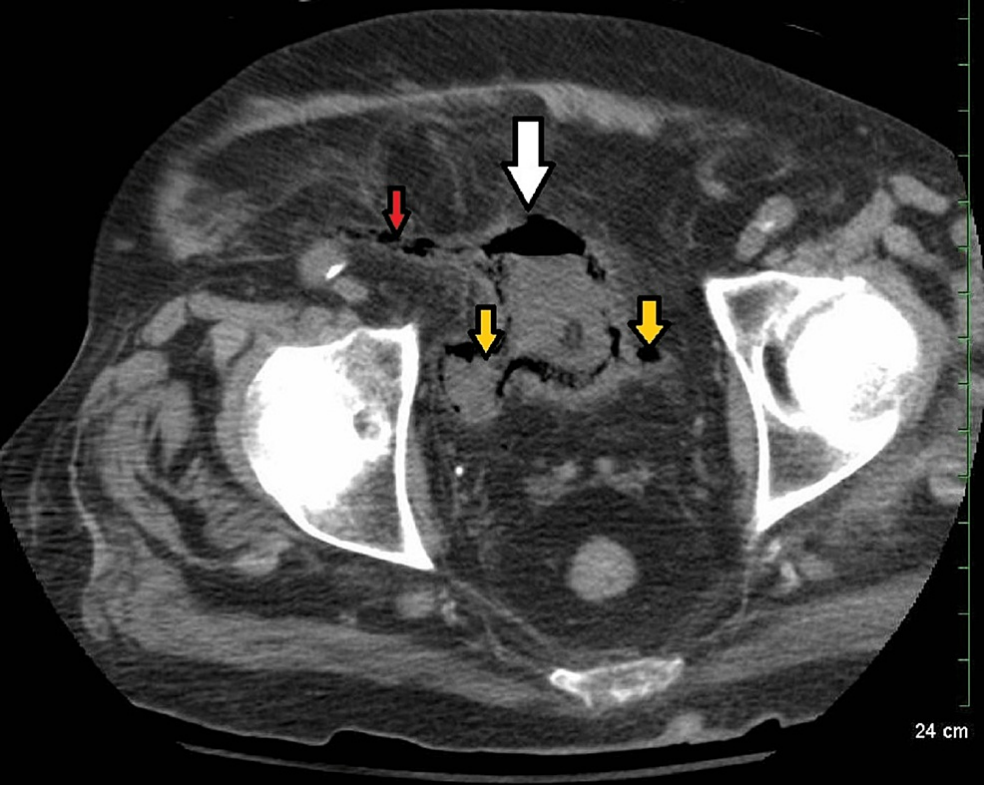
Non-enhanced CT image showing gas in the bladder (white arrow), transplanted distal ureter (red arrow), and native distal ureters (yellow arrow).

Complete blood picture (CBP) showed an increased WBC count of 20,000/mm³ with bandemia, hemoglobin of 8.1 g/dL, platelet count of 217,000/mm³, lactic acid of 5 mmol/L, blood urea nitrogen (BUN) 92 mg/dL, creatinine 3 mg/dL (baseline BUN/creatinine 31/0.76), sodium 132 mmol/L, potassium 5.8 mmol/L, and bicarbonate of 16 mmol/L. Urinalysis showed turbid, pink-red urine, WBC of more than 100 per high-power field, packed RBCs, marked bacteria, large leukocyte esterase, positive nitrate, and more than 300 mg/dL of protein with a pH of 7. An ECG showed normal sinus rhythm. A chest X-ray showed decreased lung volumes with bibasilar atelectasis and a right-sided peripherally inserted central line (PICC), with its tip near the mid-right atrium. With elevated lactic acid, hypotension, and acute renal failure, a CT abdomen without contrast was done, which showed bilateral hydronephrosis, dilated transplant kidney, with gas in all three kidneys (Figures 1-3), collecting systems, ureters (Figures 2-4), and bladder wall emphysema (Figure 4).

In the ER, he received 30 milliliters per Kilogram of fluid resuscitation per sepsis protocol. Because of chronic steroid use, he also received 100 mg of hydrocortisone followed by 50 mg of hydrocortisone every six hours. He was started on norepinephrine to maintain mean arterial pressure of 65 mm of Hg and IV meropenem, given his ESBL history [7]. He required admission to the ICU. With ongoing hematuria, his hemoglobin dropped and required one unit of packed RBCs. Immediate consultation with a urologist and nephrologist was obtained. Urology recommended emergent nephrostomy tubes for all three kidneys because of significant disease, as he was not a good surgical candidate. The patient has multiple risk factors, including septic shock at admission, immunocompromised state, acute renal failure, proteinuria, hypoalbuminemia, and high risk for major adverse cardiovascular events during surgery. 

Gupta Perioperative Estimated Risk probability for perioperative myocardial infarction or cardiac arrest is 7.8%. The American College of Surgeons National Surgical Quality Improvement Program (NSQIP) surgical risk showed 43% severe complications, 40% chance of death, 28.8% risk of developing sepsis, and 14% risk of pneumonia and cardiac complications [8]. Because of this, the family opted for conservative management with antibiotics, IV fluids, and vasopressors. Unfortunately, his hospital course further deteriorated, requiring ventilatory support 14 hours after admission. The patient's family opted for comfort care because of poor prognosis and rapid deterioration with profound sepsis requiring ventilatory support. The patient passed peacefully on the second day of admission. However, follow-up of his urine culture again grew ESBL *E. coli*.

## Discussion

The most common clinical symptoms of EPN are fever, flank pain, and pyuria. Unfortunately, there is no specific clinical presentation. The exact mechanism of gas formation and pathogenesis in EPN is unclear. However, the most commonly accepted hypothesis is the simultaneous presence of pathogenic fermenting bacteria, high serum glucose levels, and impaired tissue perfusion [9]. Diabetes mellitus and immunocompromised status are the major risk factors for EPN.

Since the 1970s, multiple radiologic classifications have been described for EPN. These classifications are based on gas in the renal parenchyma, Gerota fascia, and single or bilateral kidney involvement. The most recent classification in 2000 by Huang JJ and Tseng CC (Table 1) is a modified classification by Michaeli J et al. from 1984 [10]. 

**Table 1:** Huang JJ and Tseng CC emphysematous pyelonephritis classification of native kidneys based on CT findings.

Category	Definition	Risk of mortality
Class 1	Gas in the collecting system	0%
Class 2	Gas in renal parenchyma with no extension beyond the organ	10%
Class 3A	Gas and/or abscess in perirenal space	29%
Class 3B	Gas and/or abscess in para-renal space	19%
Class 4	Bilateral emphysematous pyelonephritis or affecting a single solitary kidney	59%

EPN in transplant kidneys is classified by Al-Geizawi SM et al. (Table 2) based on the gas extension, the severity of the infection, and renal function impairment. Our case is classified as stage 3 [11].

**Table 2:** Al-Geizawi SM et al. emphysematous pyelonephritis classification of the renal allograft.

Category	Definition	Risk of mortality
Stage 1	Gas in the collecting system	0%
Stage 2	Gas in <50% of renal parenchyma, with minimal extension to perirenal space, quickly controlled sepsis	0%
Stage 3	Gas in >50% of renal parenchyma or extensive spread to perirenal space or evidence of organ failure, uncontrolled sepsis, a non-responder shock to therapeutic measures	25%

EPN is diagnosed based on clinical and imaging findings. A plain abdominal X-ray can only detect gas 30% of the time. USG sometimes cannot clearly distinguish gas in the bowel from necrotic gas-filled kidneys, so a CT scan of the abdomen with and without contrast is the best diagnostic test that shows the presence of air and the extent of the disease [12]. Conservative management is generally preferred for classes 1 and 2 and stages 1 and 2, including parenteral antibiotic therapy and possible percutaneous drainage. Urgent endoscopic or percutaneous drainage and nephrectomy should be reserved for severe forms (classes 3 and 4 and stage 3) and those who failed conservative treatment with IV antibiotics and drainage [13].

If diagnosed early, the survival rate is around 75-85%. The presence of septic shock, bacteremia, hypoalbuminemia, proteinuria, thrombocytopenia, acute metabolic encephalopathy, disseminated intravascular coagulation (DIC), acute renal failure requiring hemodialysis, and polymicrobial infection has a poor prognosis. The presence of 2 or more of the above findings carries the highest mortality risk [14]. Severe proteinuria is an independent risk factor for poor outcomes. Our patient had septic shock, acute renal failure, and severe proteinuria. Age, gender, site of infection, and blood glucose level are not associated with mortality or poor outcome.

As EPN has no specific signs and symptoms, a high index of suspicion should be present in people with diabetes with UTIs. They are incredibly sick or slowly responding to standard IV antibiotic treatment. Early imaging should be performed to provide appropriate treatment. Once the diagnosis is confirmed, aggressive fluid resuscitation, glucose control, appropriate antibiotic use, and urgent urologic intervention (cystoscopy, stenting, percutaneous nephrostomy tube placement, or nephrectomy) should be performed to improve the symptoms. Broad-spectrum IV antibiotics should target Gram-negative bacteria, considering the patient's previous culture, sensitivities, and local antibiotic resistance patterns.

Historically, emergent nephrectomy has been the preferred treatment of choice for EPN. The success rate with conservative management and percutaneous drainage is around 60-80% [15]. The success rate with nephrectomy is around 70-90%. Bilateral nephrectomies are associated with extremely high mortality but may have shorter hospital stays. However, they also require lifelong renal replacement therapy, which has complications [10].

Our patient presented with septic shock and was immunocompromised, diabetic, and had class 4 EPN. Unfortunately, he was not a surgical candidate because of his very high cardiovascular risk.

## Conclusions

EPN is a rare, life-threatening complicated UTI typically seen in diabetic patients. A high index of suspicion should be present for EPN in patients with known risk factors. CT abdomen with and without contrast is the definitive diagnostic test. Management is with aggressive fluid resuscitation, antibiotics, and early urologic interventions. Mortality rates are very high, just with aggressive medical management alone.
